# Primary Investigation for the Mechanism of Biatractylolide from *Atractylodis Macrocephalae Rhizoma* as an Acetylcholinesterase Inhibitor

**DOI:** 10.1155/2016/7481323

**Published:** 2016-08-24

**Authors:** Yong-Chao Xie, Ning Ning, Li Zhu, Dan-Ning Li, Xing Feng, Xiao-Ping Yang

**Affiliations:** ^1^Department of Pharmacy, School of Medicine, Hunan Normal University, Changsha, Hunan 410013, China; ^2^The First Affiliated Hospital, Hunan Normal University, 61 West Jiefang Road, Changsha, Hunan 410005, China

## Abstract

Biatractylolide was isolated from ethyl acetate extract of dried* Atractylodis Macrocephalae Rhizoma* root by multistep chromatographic processing. Structure of biatractylolide was confirmed by ^1^H-NMR and ^13^C-NMR. The IC_50_ on acetylcholinesterase (AChE) activity was 6.5458 *μ*g/mL when the control IC_50_ value of huperzine A was 0.0192 *μ*g/mL. Molecular Docking Software (MOE) was used to discover molecular sites of action between biatractylolide and AChE protein by regular molecular docking approaches. Moreover, biatractylolide downregulated the expression of AChE of MEF and 293T cells in a dose-dependent manner. These results demonstrated that the molecular mechanisms of inhibitory activities of biatractylolide on AChE are not only through binding to AChE, but also via reducing AChE expression by inhibiting the activity of GSK3*β*.

## 1. Introduction

Alzheimer's disease (AD) is an age-related neurodegenerative disease, which is the most frequent and predominant cause of dementia in the elderly, provoking progressive cognitive decline, psychobehavior disturbances, memory loss, the presence of senile plaques, neurofibrillary tangles, and the decrease in cholinergic transmission [1, 2]. Many risk factors have participated in AD pathogenesis, but studies have demonstrated that GSK3*β* played the most critical role in this disease conditions [3, 4]. Abnormal activity of GSK3*β* not only led to A*β* generation and aggregation [5], tau protein phosphorylation, and high mitochondrial dysfunction [6], but also upregulated the expression of AChE in AD [7]. Recently, two major hypotheses: the cholinergic hypothesis and the amyloid cascade hypothesis, have been proposed to elucidate the molecular mechanism of AD pathogenesis. In order to treat and prevent AD, most pharmacological research has focused on AChE inhibitors to alleviate cholinergic deficit and improve neurotransmission. AChE plays a significant role in the termination of nerve impulse transmission at the cholinergic synapses by rapid hydrolysis of acetylcholine (ACh). AChE inhibitors can increase the cholinergic transmission by blocking the degradation of ACh and are therefore considered to be a promising approach for the treatment of Alzheimer's disease [8]. The roots of* Atractylodis Macrocephalae Rhizoma* have been widely used in traditional Chinese medicine due to their antioxidative and preventive effects of AD. Biatractylolide is an active component existing in Atractylodis Macrocephalae Rhizome. This small molecule has a symmetrical structure which has a novel double sesquiterpene esters. Reference [9] reported that biatractylolide could slow down the isolated guinea pig right atrium heart rate and reduce shrinkage force. However, this effect could be offset by atropine, indicating that biatractylolide might inhibit cholinesterase effect. Our previous study showed that biatractylolide can significantly reduce the activity of AChE in mice brain and improve the syndrome of mouse dementia induced by aluminum trichloride [10]. However, the molecular mechanisms of biatractylolide on anti-AD remained unclear. In this study, extracted biatractylolide from Chinese traditional medicine Atractylodis Macrocephalae Rhizome was applied to explore its molecular mechanisms of inhibiting AChE, providing a theoretical basis for of its clinical application on anti-AD syndrome.

## 2. Materials and Methods

### 2.1. Materials

Biatractylolide was isolated from ethyl acetate extract of dried* Atractylodis Macrocephalae Rhizoma* root by multistep chromatographic processing. Structure of biatractylolide was confirmed by ^1^H-NMR and ^13^C-NMR. Lithium chloride was purchased from Sigma Chemicals (St. Louis, MO, USA). Anti-AChE antibody (H-134), anti-GSK3*β* (H-76) antibody, and GAPGH (FL-335) antibody were purchased from Santa Cruz Biotechnology Inc (Santa Cruz, CA, USA) and were diluted by 1 : 1000 when used. Complementary DNA (cDNA) encoding full-length human AChE-S (hAChE-S) and human AChE-R (hAChE-R) cloned into the pL5CA expression vector were gifts from Dr. Hermona Soreq at the Hebrew University of Jerusalem. The correct sequences of all cDNA clones were verified by automated DNA sequencing analysis.

### 2.2. Extraction and Isolation

The air-dried* Atractylodis Macrocephalae Rhizoma* (15 kg) were extracted with EtOAc at room temperature for 7 days three times. The solvent was evaporated under reduced pressure to obtain a dark green extract (275 g). The extract was dissolved and partitioned between petroleum ether and ethyl acetate. The petroleum ether portion was subjected to a silica gel column and eluted with a petroleum ether–ethyl acetate gradient system (PE–EtOAc 20 : 1, 10 : 1, 10 : 2, 10 : 3). The portion of 10 : 1 was subjected to repeated column chromatography with silicagel (petroleum ether-acetone, 20 : 1 to 5 : 2) and further purification by preparative TLC to obtain biatractylolide (480 mg).

### 2.3. Molecular Docking Studies

We drew out biatractylolide dimensional structure by ChemDraw software and then imported structure into Chem3D, and biatractylolide molecules energy optimized by MM2 force field, saved as mol2 format; finally, biatractylolide molecules had the hydrogenation and energy optimization by MOE software. We searched three-dimensional structure of AChE targets from the protein database and then found the key of active amino acid residues for molecular docking into MOE software. We had analyzed the relationship between active amino acid residues and biatractylolide, predicted interaction between biatractylolide and AChE protein target, and elucidated chemical structural foundation of biatractylolide as AChE inhibitor.

### 2.4. Determination of AChE Inhibition Activities

AChE inhibitory activity was measured by using an assay described by Ellman et al. [11] along with the modifications described by Hostettmann et al. [12]. Briefly, the sample (20 *µ*L) (biatractylolide was dissolved in DMSO and diluted with PBS to 12 *μ*g/mL, 10 *μ*g/mL, 8 *μ*g/mL, 6 *μ*g/mL, 4 *μ*g/mL, 3 *μ*g/mL, 2 *μ*g/mL, and 1 *μ*g/mL; huperzine A was dissolved in methyl alcohol and diluted with PBS to 0.065 *μ*g/mL, 0.050 *μ*g/mL, 0.035 *μ*g/mL, 0.025 *μ*g/mL, 0.020 *μ*g/mL, 0.015 *μ*g/mL, 0.010 *μ*g/mL, and 0.005 *μ*g/mL), along with the buffer (140 *µ*L) and 0.28 U/mL AChE (15 *µ*L), and the mixture were incubated at 37°C for 15 min. The time at which the first enzyme addition was performed was considered as time zero. After the 15 min incubation, 2 mM DTNB (10 *µ*L) and 15 mM ATCI (10 *µ*L) were added, and the final mixture was incubated at room temperature for 30 min. The absorbance of the mixture was measured at 402 nm by using a microplate reader (Synergy H4 Hybrid Multi-Mode Microplate Reader, BioTek, Winooski, VT, USA). A control mixture was prepared by using 20 *µ*L of PBS instead of the biatractylolide sample, with all other procedures similar to those used in the case of the sample mixture. The percentage inhibition of enzyme activity was calculated by comparison with the negative control: % = [(*A*_0_ − *A*_1_)/*A*_0_] × 100, where *A*_0_ was the absorbance of the blank sample and *A*_1_ was the absorbance of the sample. Tests were carried out in triplicate. The inhibition of the enzyme activity was expressed as IC_50_ (the concentration of the sample required to inhibit 50% of enzyme), which was calculated by a curve fitting analysis of the dose-effect relationship, used by OriginPro 9.0.

### 2.5. Cell Culture and Transfection

HEK293T cells were grown in high-glucose Dulbecco's modified Eagle Medium (DMEM) (Sigma, Louis. MO, USA) supplemented with 10% fetal bovine serum (FBS) (GIBCO-BRL, Gaithersburg, MD, USA), 2 mM L-glutamine (Corning, Manassas, VA, USA) and nonessential amino acids, and 1% penicillin. Cells were trypsinized and reseeded in culture plates one day before transfection. Cells were grown at 37°C in 5% CO_2_. HEK293T cells were transfected with Lipofectamine 2000 when cell confluency was 70%. Mouse embryonic fibroblast (MEF) cells were cultured in Dulbecco's Modified Eagle's Medium (Invitrogen, Carlsbad, CA, USA) with 15% fetal bovine serum (Thermo Scientific) and 4 mM L-glutamine and nonessential amino acids (Invitrogen). Cells were trypsinized and reseeded in culture plates 1 day before transfection. Transfection was performed with Lipofectamine LTX and PlusTM reagent (Invitrogen) when cell confluency was 70% according to the manufacturer's instructions.

### 2.6. Western Blotting

Cultured cells were washed twice with ice-cold PBS, scraped off the plate, centrifuged at 1500 rpm, and cleared of cellular debris by centrifugation (12,000 rpm, 10 min, 4°C). Protein concentration was determined using a BCA protein assay kit (Pierce). Cell lysates (100 mg protein each) were separated by 4–12% SDS-PAGE (sodium dodecyl sulfate-polyacrylamide gel electrophoresis) electrophoresis and electroblotted to nitrocellulose membrane (Bio-Rad). The membranes were blocked in TBST containing 5% nonfat dry milk for 1 h at room temperature. Blotted membranes were probed with their respective primary antibodies rotating at 4°C overnight. The membranes were washed three times in TBST buffer and probed with secondary antibody (Alexa Fluor 680 goat anti-rabbit IgG or IRDye 800-conjugated Affinity Purified Anti-Mouse IgG, resp.) at room temperature for 1 h. Membranes were then washed three times in TBST buffer and direct infrared fluorescence detection was performed with a Licor Odyssey Infrared Imaging System [13]

### 2.7. Statistical Analysis

Data are presented as the mean ± SD, and significant differences between treatment groups were determined using the unpaired Student's *t*-test. Differences were considered statistically significant when *p* < 0.05.

## 3. Results and Discussion

### 3.1. Extraction and Separation

**Figure 1:**
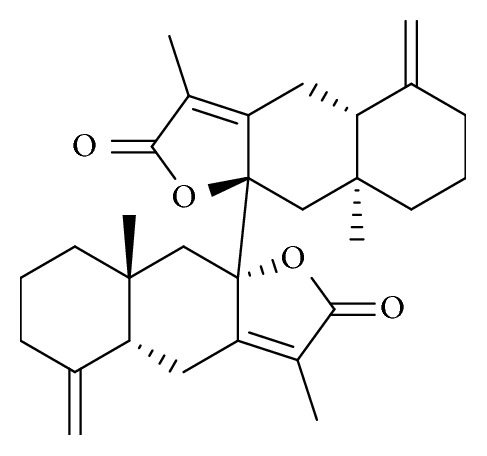
Chemical structures of biatractylolide. Biatractylolide is a kind of internal symmetry double sesquiterpene novel ester compound.

Biatractylolide was isolated from ethyl acetate extract of dried* Atractylodis Macrocephalae Rhizoma* root by multistep chromatographic processing. Chemical structure of biatractylolide was confirmed by ^1^H-NMR and ^13^C-NMR. The spectral data is in agreement with the literature [14] (see Figure 1).

### 3.2. Molecular Docking Studies

**Figure 2:**
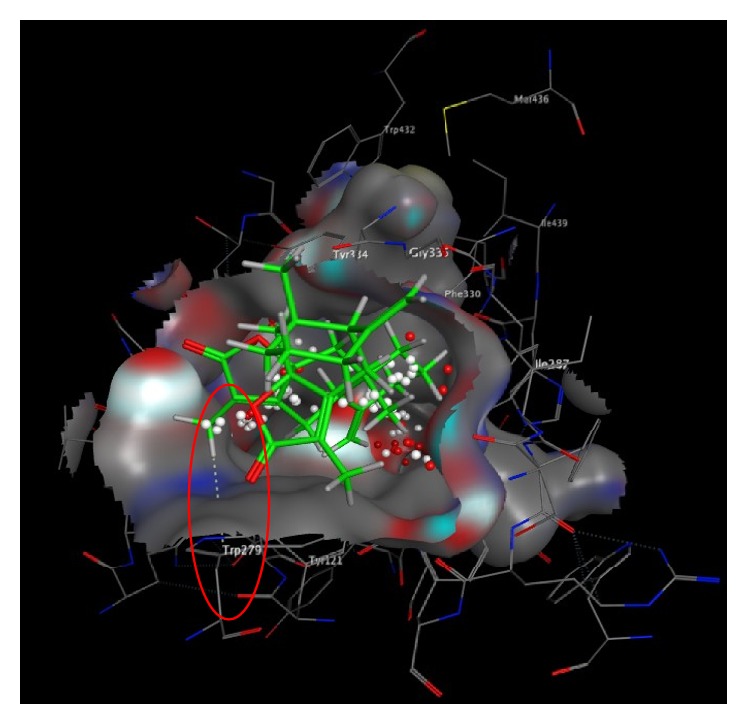
Molecular docking between biatractylolide and PAS active site. The figure showed biatractylolide combined with AChE protein important active amino acid residues Trp279 (PAS active site) by H-*π* (3.31 Å).

**Figure 3:**
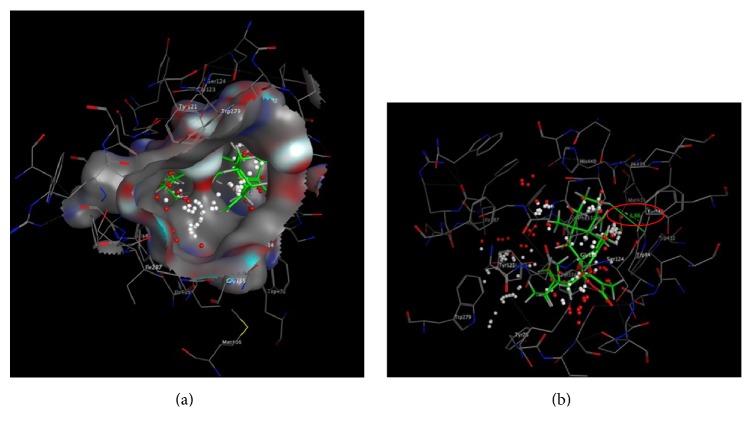
Molecular docking between biatractylolide and CAS active site. (a) showed biatractylolide entire molecule passed through the narrow “aromatic gorge” into the AChE protein activity pocket bottom. (b) showed biatractylolide also combined with Trp84 (CAS active site amino acid residues) by H-*π* (3.88 Å).

We found a number of important active amino acid residues on PAS and CAS activity center: PAS: Trp279, Tyr70, and Tyr334; CAS: Trp84, IIe439, IIe287, and Tyr121. MOE molecular docking showed that biatractylolide combined with AChE protein important active amino acid residues Trp279 (PAS active site) by H-*π* (3.31 Å) (see Figure 2). Biatractylolide entire molecule passed through the narrow “aromatic gorge” into the AChE protein activity pocket bottom (Figure 3(a)). Biatractylolide also combined with Trp84 (CAS active site amino acid residues) by H-*π* (3.88 Å) (Figure 3(b)). The results indicated that biatractylolide could be combined with AChE protein by a dual target binding. However, it bound the CAS active site with more stabilities.

Biatractylolide molecule is a large molecule with steric hindrance. It has much chiral carbon, and it has no conjugate rigid structure such as aromatic ring, so biatractylolide molecular after conformation twisting could smoothly pass through the AChE aromatic gorge into interior of the protein activity pocket and then it had effect with Trp84 and Trp279, two active amino acid residues, which may be the reason it has AChE inhibition.

### 3.3. Determination of AChE Inhibitory Activities

**Figure 4:**
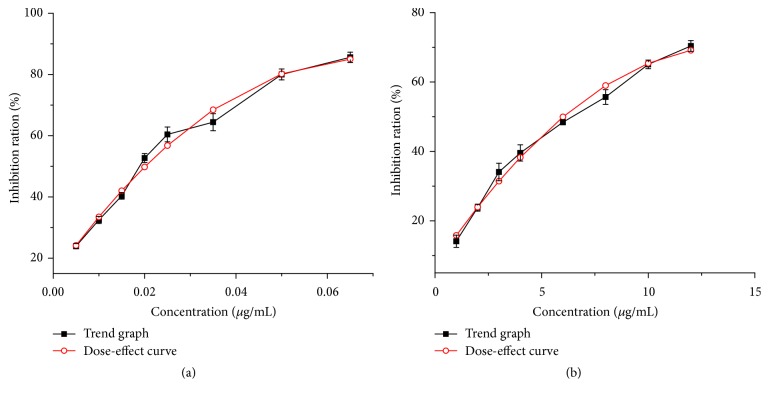
Different concentrations of huperzine A and biatractylolide corresponding AChE inhibition. The curve fitting of huperzine A was *y* = −15411.47*x* ^2^ + 2092.92*x* + 14.09 (*R* ^2^ = 0.9824, IC_50_ = 0.0192 *μ*g/mL = 0.0793 *μ*M). The curve fitting of biatractylolide was *y* = −0.33*x* ^2^ + 9.14*x* + 6.99 (*R* ^2^ = 0.9874, IC_50_ = 6.5458 *μ*g/mL = 14.1685 *μ*M).

In our ongoing search for biatractylolide AChE inhibitors, it was found that biatractylolide mildly inhibited the AChE activity with an IC_50_ value of 6.5458 *μ*g/mL (see Figure 4) when huperzine A showed AChE inhibitory activity with an IC_50_ value of 0.0192 *μ*g/mL.

### 3.4. AChE Protein Characterization

**Figure 5:**
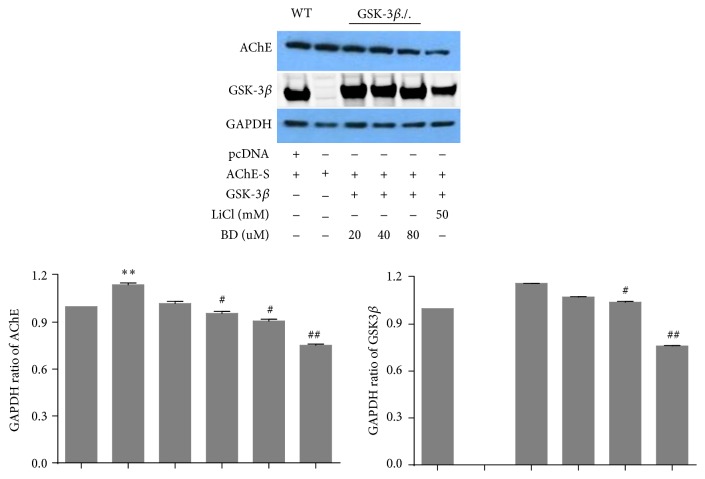
AChE protein characterization of MEF. The figure represents western blotting of AChE and GSK-3*β* after MEF cell lines were treated by BD (biatractylolide) and LiCl. GAPDH was included as a loading control. The ratio of different proteins to GAPDH was calculated by the band density of western blots of each cell line using Image J software. ^#^ *p* < 0.05 versus Control Group, ^##^ *p* < 0.01 versus Control Group, ^*∗*^ *p* < 0.05 versus WT Group, and ^*∗∗*^ *p* < 0.01 versus WT Group.

**Figure 6:**
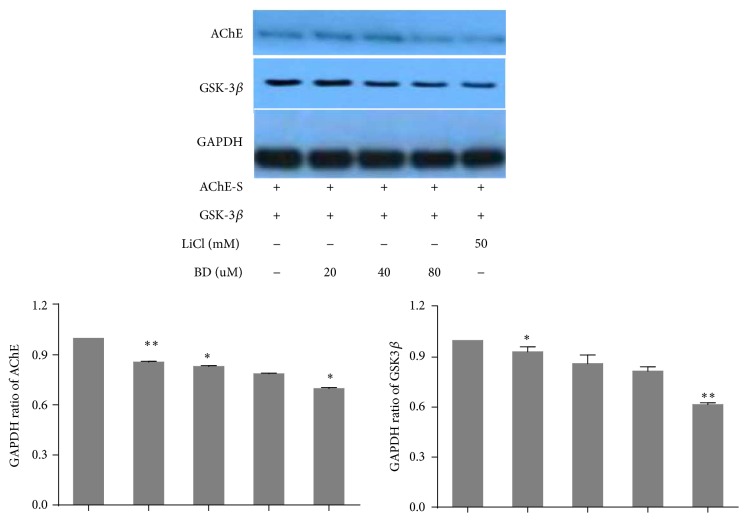
AChE protein characterization of 293T. The figure represents western blotting of AChE and GSK-3*β* after MEF cell lines were treated by BD (biatractylolide) and LiCl. GAPDH was included as a loading control. The ratio of different proteins to GAPDH was calculated by the band density of western blots of each cell line using Image J software. ^*∗*^ *p* < 0.05 versus Control Group; ^*∗∗*^ *p* < 0.01 versus Control Group.

Figure 5 showed wild type MEF cells which was transfected with the AChE-S expressed AChE protein, and so did knocked out GSK3*β* gene MEF cells, indicating the success of the transfection. We added different concentrations of biatractylolide and 50 mM LiCl (a kind of specific inhibitor for GSK3*β* which is reported to stop the expression of AChE-S in apoptosis process [3] by inhibiting GSK3*β*) after MEF cells which knocked out GSK3*β* transfected heterologous gene GSK3*β* and AChE-S. The result showed that AChE expression gradually reduced with increased biatractylolide concentrations. Moreover, AChE had low expression after adding LiCl. However, the expression levels of AChE are still not high after being transfected with AChE-S in MEF cells. In order to further confirm our results, we chose 293T cell whose transfection efficiency is higher than MEF cells; we transfected AChE-S and GSK3*β* into 293T cells directly (Figure 6). As the biatractylolide concentration increased, we observed that biatractylolide Control Group had high expression of GSK3*β* and AChE. However, the expression of AChE was gradually reduced, so we hypothesized that biatractylolide downregulated expression levels of AChE by inhibiting GSK3*β*.

The incidence of AD is increasing with the acceleration of global ageing. It is estimated that the world will have at least 48 million AD patients in 2024, while China is one of the countries most affected by aging. Thus, the development of new anti-AD drugs is of great significance.

AChE inhibitors combined with AChE to inhibit the hydrolysis of AChE activity, increasing ACh content between synapses in the brain, and then improving the cognitive ability of patients with AD. Currently most of available anti-AD drugs are AChE inhibitors, such as donepezil and rivastigmine, but they have severe side effects which limited the clinical application [15]. The fact that naturally occurring compounds from plants are considered to be a potential source of new inhibitors has led to the discovery of an important number of secondary metabolites and plant extracts with the ability of inhibiting the enzyme AChE. Sepsova et al. [16] separated N-methyl shiva alkali from* lotus* which was a noncompetitive inhibitor of AChE with an IC_50_ value of 1.5 *μ*g/mL. Barak et al. [17] obtained six berberine compounds from the Chinese traditional medicine* Coptis* which had the function of the nerve protection and improvement of cognition and confirmed strong AChE inhibitory activity with an IC_50_ value 1.44~1.80 *μ*g/mL. Berg et al. [18] isolated Macluraxanthone from* Maclura pomifera* which was a noncompetitive AChE inhibitor with IC_50_ value 8.47 *μ*g/mL. All these results have shown a strong prospect using plant sources to extract and develop anti-AD drugs.

Molecular docking is widely used in the development of AChE inhibitors [19]. Amino acid residues Trp 279, Tyr 70, and Tyr 334 are negatively charged and located in PAS [20, 21]. Compounds profenamine, BW284C5, and fasciculin could have effects with PAS active site [22]. The occupation and mutation of Trp279 site seriously affected the affinity of enzyme and substrate, and the enzyme activity. Our molecular docking studies showed biatractylolide to interact with Trp279 via H-*π* (3.31 Å) role, indicating that biatractylolide could affect enzyme activity by occupying the site.

Trp84 located in CAS catalytic center; study has shown that ACh quaternary ammonia nitrogen atoms could have effect with Trp84 ring benzene on the amino acid residues, and the distance between them is 4.8 Å [23]. Biatractylolide lactone ring methyl hydrogen atom interacts with Trp84 ring benzene on the amino acid residues, so biatractylolide is a two-site AChE inhibitor.

The apoptosis rate of nerve cells in AD patients' brains is higher and so is AChE activity in pathologic region. In addition, AChE facilitates the poisonousness precipitation of A*β* sample. Among the three AChE subtypes, only AChE-S is capable of hydrolyzing AChE [24]. Transgenic mouse overexpressing AChE-S bears apparently more dynamic activity in catalyzing the hydrolyzation of ACh. The obvious increase in expressing caspase-3 and apoptotic protein in nerve cells justifies that the overexpression of AChE-S facilitates the apoptosis of nerve cells [25]. Studies [26] have showed that transfecting N-terminal AChE-S to cortical neurons can lead to apoptosis, mitochondria damage, overexpression of caspase-3 and GSK3*β* activation, and accelerating highly phosphorylation of tau. Moreover, by inhibiting GSK3*β* and having apoptosis inhibitory protein Bcl2 overexpressed, survival rate of nerve cells improves. These facts demonstrated that AChE-S could be used as a target spot in developing anti-AD drugs. Our previous study proved that biatractylolide cures AD model rats. In this study, we are interested in investigating the molecular mechanism underlying inhibition of AChE expression. These results have showed that biatractylolide inhibits the expression of AChE, where AChE-S and GSK3*β* genes are transfected to two kinds of engineering cell aiming at directly exploring the effects of biatractylolide on the expression of AChE.

MEF cells are one of the mouse embryonic fibroblasts stem cell lines with properties of easy working on and physiological relevance with AD. Therefore, we used MEF as a working cell model to investigate foreign gene function and cell signaling pathway along with determining the effects of this compound. We transfected foreign AChE-S genes and GSK3*β* genes to MEF cells during the course of adding 50 mM LiCl and various concentrations of biatractylolide. Biatractylolide reduces the expression of AChE by inhibiting GSK3*β*.

## 4. Conclusion

Our study demonstrated that biatractylolide can inhibit the enzyme activity of AChE and downregulate the expression of AChE in a dose-dependent manner. Biatractylolide has a great potential to be used as an anti-AD drug.
